# Acetylcholinesterase: The “Hub” for Neurodegenerative Diseases and Chemical Weapons Convention

**DOI:** 10.3390/biom10030414

**Published:** 2020-03-07

**Authors:** Samir F. de A. Cavalcante, Alessandro B. C. Simas, Marcos C. Barcellos, Victor G. M. de Oliveira, Roberto B. Sousa, Paulo A. de M. Cabral, Kamil Kuča, Tanos C. C. França

**Affiliations:** 1 Institute of Chemical, Biological, Radiological and Nuclear Defense (IDQBRN), Brazilian Army Technological Center (CTEx), Avenida das Américas 28705, Rio de Janeiro 23020-470, Brazil; barcellos.marcos@eb.mil.br (M.C.B.); victor.oliveira@eb.mil.br (V.G.M.d.O.); roberto.barbosa@eb.mil.br (R.B.S.); paulo.cabral@eb.mil.br (P.A.d.M.C.); 2 Walter Mors Institute of Research on Natural Products (IPPN), Federal University of Rio de Janeiro (UFRJ), CCS, Bloco H, Rio de Janeiro 21941-902, Brazil; 3 Department of Chemistry, Faculty of Science, University of Hradec Králové, Rokitanskeho 62, 50003 Hradec Králové, Czech Republic; 4 Laboratory of Molecular Modelling Applied to Chemical and Biological Defense (LMACBD), Military Institute of Engineering (IME), Praça General Tibúrcio 80, Rio de Janeiro 22290-270, Brazil

**Keywords:** acetylcholinesterase, Alzheimer’s disease, nerve agents, Chemical Weapons Convention

## Abstract

This article describes acetylcholinesterase (AChE), an enzyme involved in parasympathetic neurotransmission, its activity, and how its inhibition can be pharmacologically useful for treating dementia, caused by Alzheimer’s disease, or as a warfare method due to the action of nerve agents. The chemical concepts related to the irreversible inhibition of AChE, its reactivation, and aging are discussed, along with a relationship to the current international legislation on chemical weapons.

## 1. Introduction

**Figure 1:**
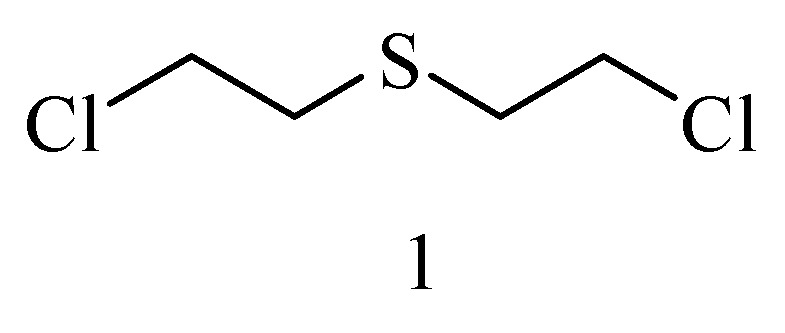
Sulfur mustard HD1.

The use of chemical weapons (CW) is quite common throughout history. Over time, the use of incendiaries, darts, spears, and arrows impregnated with poisons from different organisms, animals, and plants, the lethality of substances used for warfare have been dramatically improved. Despite international agreements already in existence by the end of 19th century, these did not inhibit the large-scale use of CW during World War I (WWI) (1914–1918), when millions of casualties were provoked mainly by phosgene, chlorine, and, particularly, sulfur mustard (HD **1**) (Figure 1). This compound, due to its physicochemical characteristics, is known as “area denial weapon”, precluding further access to the place where it has been deployed [1].

**Figure 2:**
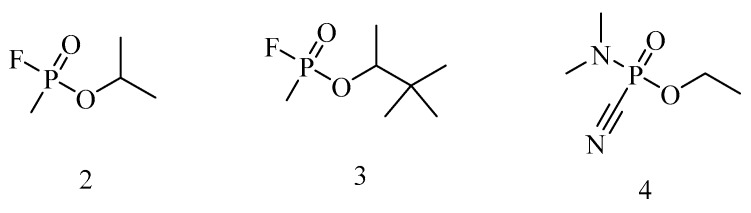
G-Agents.

After WWI and more actively during WWII, while looking for new pesticides, German scientists developed a series of pentavalent phosphorus compounds that contained a leaving group such as a halogen. They discovered that these compounds were very toxic, and capable of inhibiting acetylcholinesterase (AChE). These findings resulted in the weaponization of the first nerve agents, called also G-agents, which included sarin **2** (GB), soman **3** (GD), and tabun **4** (GA) (Figure 2). Such CW were produced and stockpiled, but never used during WWII [1,2,3,4,5,6,7,8,9,10,11].

**Figure 3:**
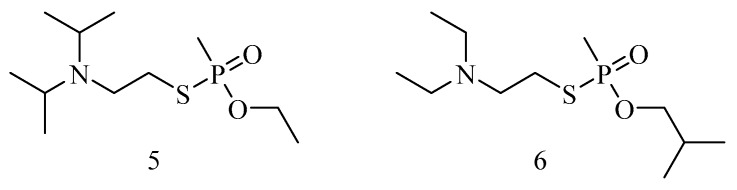
V-Agents.

Later, during the 1950s, aiming to produce more effective pesticides, groups in the former Soviet Union, Great Britain, Sweden, and the United States developed other organophosphorus compounds which were more toxic and persistent (less volatile) than G-agents. These new chemicals were then named V-agents, which are phosphonothioates (**5** and **6**, Figure 3) [4,12,13,14,15]. Like sulfur mustard HD **1**, these compounds can be similarly regarded as “area denial weapons” [2,8].

Although they were not used in the World Wars, the international community could soon testify their potential as Weapons of Mass Destruction (WMD) when, in the 1980s, Saddam Hussein’s troops used chemical artillery containing G-agents, GB **2** and GA **4**, along with sulfur mustard HD **1,** against civilian and military targets during the Iran–Iraq war. The tragic events in the Kurdish city of Halabja show how abhorrent can be the use of these substances as warfare agents [16,17,18]. In 1994 and 1995, Japan was stricken by two episodes when GB **2** was employed in the terrorist actions carried out by the sect *Aum Shinrikyo*, provoking casualties among civilians and first responders [4,19,20,21,22].

Although many papers have dealt with chemical warfare over the years, the use of CW is currently a pressing subject. Recently, chemical warfare agents have made headlines due to their alleged deployment in the Civil War ongoing in Syria (2011–) and as means of murdering the North Korean national Kim Jun Nam at the Kuala Lumpur International Airport in 2017. A report from Malaysian authorities confirmed that VX was used by the perpetrators [4,23,24,25,26,27,28,29,30,31,32,33,34,35,36,37,38,39,40,41,42].

**Figure 4:**
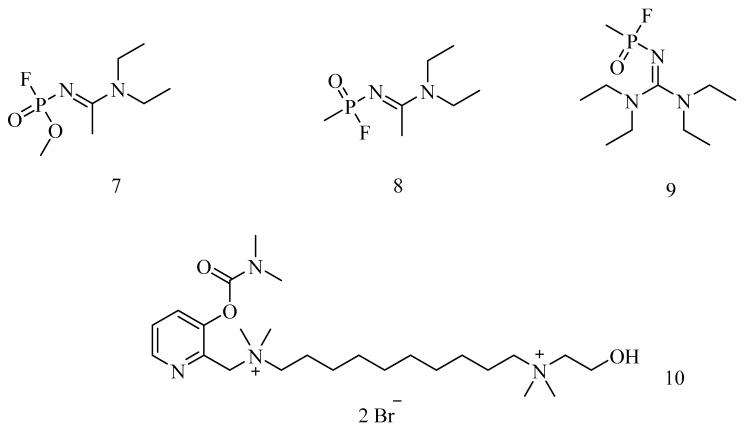
Some considered structures for Novichok Agents according to the Chemical Weapons Convention (CWC).

In 2018, events that occurred in Salisbury and Amesbury, Great Britain, where a former Russian agent and other people were poisoned with an “unknown, high-purity grade military agent” with possible neurotoxic effects, prompted discussions about the so-called “Novichok” agents. These then elusive compounds are predicted to act similarly to known nerve agents, but with higher toxicity and different physicochemical properties, for example, being solid at room temperature. After discussions on the actual structures of the Novichok agents, the most important agreement on chemical disarmament, the Chemical Weapons Convention (CWC), has acknowledged four novel scaffolds in its “Annex on Chemicals”, which are to be entered into force in June 2020. Figure 4 depicts examples of these compounds (**7**–**10**) [4,43,44,45,46,47,48,49,50].

From pharmacological and toxicological points of view, the biological targets of classical (G- and V-) and “Novichok” nerve agents are the cholinesterases, a class of enzymes involved in a myriad of biological processes, such as respiration, cognition, and drug metabolism. Cholinesterases are found in two isoforms, AChE (EC 3.1.1.7) and butyrylcholinesterase (BChE), EC 3.1.1.8. Compounds that can inhibit cholinesterases may impact the body positively or negatively and, therefore, be either explored as method of warfare or therapeutically [51,52,53,54,55,56,57,58,59,60,61,62,63,64,65,66,67].

Herein, we review the inhibition of cholinesterases and their outcome and how these chemical reactions can be subject of interest in the treatment of neurodegenerative diseases, like Alzheimer’s disease (AD) or as method of warfare, which is forbidden by the CWC.

## 2. The Chemical Weapons Convention

**Chart 1:**
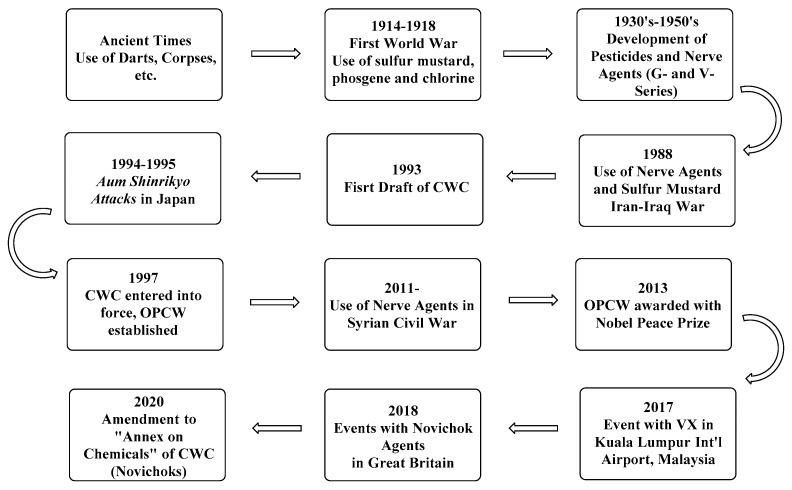
Non-comprehensive timeline of the CWC and use of chemical warfare (CW).

After many international agreements have sought to forbid the use of toxic chemicals as a mode of warfare, the Chemical Weapons Convention (CWC) entered into force in 1997, paving the way for the creation of the Organization for the Prohibition of Chemical Weapons (OPCW), the international watchdog responsible for overseeing the implementation of the CWC. The headquarters are located in The Hague, The Netherlands. As of January 2020, there are 193 signatories or State Parties to the CWC. In accordance to OPCW website, “98% of the global population lives under the protection of the Convention” and “97% of the chemical weapons stockpiles declared by possessor States have been verifiably destroyed” [1,3]. A short timeline of events using chemical warfare agents and the CWC can be devised. Although OPCW has made efforts throughout years to eliminate CW, which have granted it the Nobel Peace Prize in 2013 [1,2,6,68], the world has witnessed their recurrent use in conflicts or terrorism (Chart 1).

In the preamble of the CWC, the State Parties are called out to ensure “that the complete and effective prohibition of the development, production, acquisition, stockpiling, retention, transfer and use of chemical weapons, and their destruction, represent a necessary step towards the achievement of these common objectives” [1,2,3].

The current text of CWC has the “Annex on Chemicals” section. There, compounds considered as chemical warfare agents or precursors are listed, along with the toxins saxitoxin and ricin. Chemicals are divided in Schedules (1 to 3), accordingly to the possibility of peaceful application [1,3,4]. Schedules are subdivided in two parts, A and B, being A for toxic chemicals and B for precursors.

**Figure 5:**
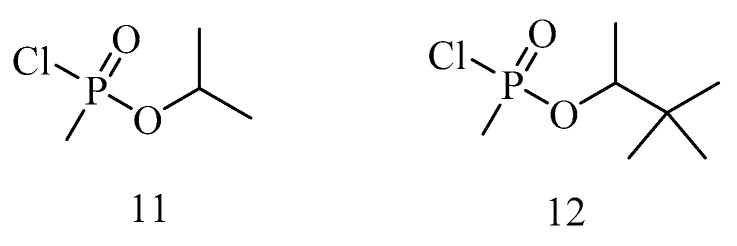
Chlorosarin and chlorosoman.

Schedule 1 list toxic chemicals which have been developed only for or used as CW. Given their toxicity and virtually no peaceful applications, they pose as a risk to the implementation of the CWC. After the terrorist events in Great Britain in 2018, Schedule 1 will receive an amendment including the “Novichok” agents, which will be entered into force in next June [48]. Compounds described in Figure 1, Figure 2, Figure 3 and Figure 4 are Schedule 1A chemicals. Examples of Schedule 1B chemicals are chlorosarin **11** and chlorosoman **12**, precursors to GB **2** and GD **3**, respectively (Figure 5) [3,4].

**Figure 6:**
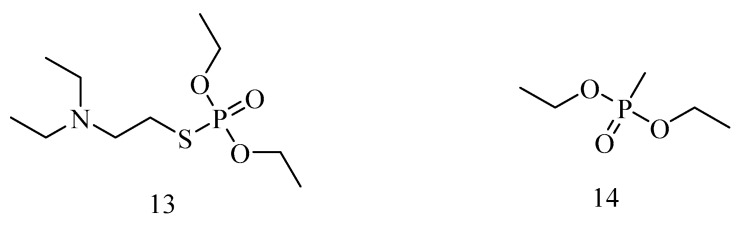
Amiton and Diethyl methylphosphonate (DEMP).

Schedule 2 contains toxic chemicals that have relevant industrial production, like the precursors for the synthesis of pesticides or medicines for example. Similar to Schedule 1, it is divided in two parts. Amiton (VG, **13**), a toxic chemical related to VX (5) is an example of Schedule 2A chemical. Diethyl methylphosphonate (DEMP, **14**), is an example of Schedule 2B chemical, as it can be precursor for toxic nerve agents, but also flame-retardant materials (Figure 6) [3,4].

**Figure 7:**
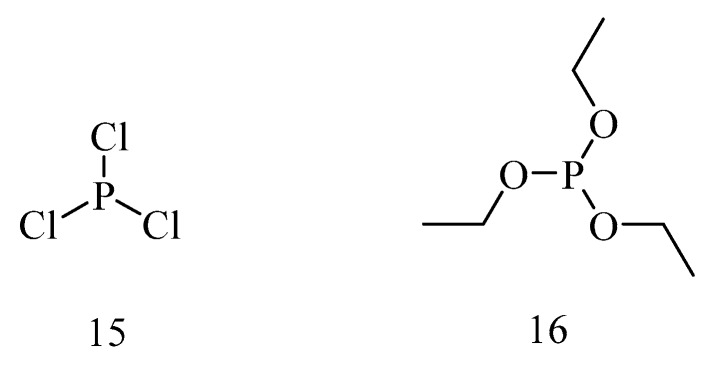
Phosphorus trichloride and triethyl phosphite.

Schedule 3 lists chemicals that are usually manufactured in large amounts for peaceful applications. Notwithstanding, they could be employed either as CW or precursors for CW listed in Schedules 1 and 2. Schedule 3 is also divided in two parts. Part A lists toxic compounds, such as phosgene, and Part B refers to precursors, which are manufactured in large scale for peaceful uses, e.g., phosphorus trichloride **15** and triethyl phosphite **16** (Figure 7) [3,4].

**Scheme 1:**
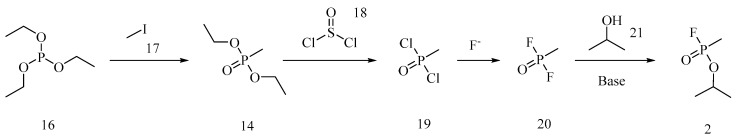
Synthetic relationship of scheduled chemicals.

Although synthetic relationships are not the criteria for the Schedule Chemicals, Scheme 1 illustrates a possible synthetic route for GB **2**, a Schedule 1A chemical, from triethyl phosphite **16**, a Schedule 3B chemical. Michaelis-Arbuzov reaction of **16** with iodomethane **17** affords DEMP (**14**, Schedule 2B chemical). DEMP may be then chlorinated using thionyl chloride **18**, a Schedule 3B chemical, yielding methylphosphonyl dichloride (MPDC, **19**), another Schedule 2B chemical. MPDC is then reacted with a source of fluoride, generating methylphosphonyl difluoride (MPDF, **20**), also a Schedule 2B chemical. Reaction of MPDF with isopropanol **21** in presence of a base, affords the toxic Schedule 1 chemical. There is comprehensive literature on the synthesis of organophosphorus compounds and researchers must read carefully the CWC, in the OPCW website (www.opcw.org) to learn more on the international legislation [1,2,3,4,69,70,71,72,73].

## 3. Cholinesterases

Chemistry is ubiquitous, its principles and applications underlie innumerous biological processes, as is the case with cholinesterases, key enzymes in the metabolism of different species. This class of enzyme in superior animals is present in two different isoforms, as aforementioned, AChE and BChE. AChE is involved in the regulation of neurotransmission processes (parasympathetic neurotransmission), being important for (but not limited to) cognition and respiration, for example. BChE is found in plasma, being responsible for hydrolysis of different esters, property that has been studied to understand a series of metabolic events or for treatment of cocaine addiction, for example. Furthermore, it has been extensively studied due to its stoichiometric reaction with organophosphorus compounds, which indicates that it is not only a biomarker of exposure to these toxic chemicals but also a potential treatment for intoxication, acting as bioscavenger of nerve agents [61,62,63,64,65,66,67,74,75,76,77,78,79,80,81,82,83].

**Figure 8:**
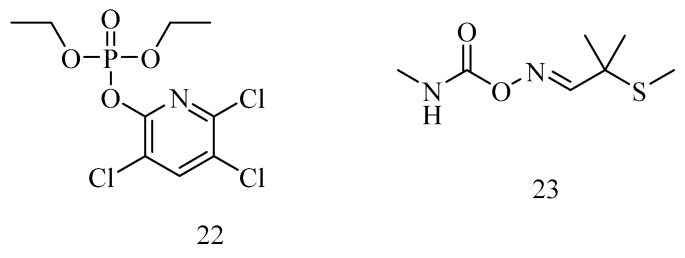
Chlorpyrifos and aldicarb.

Cholinesterases are serine-estearases, which means that their activity relies on a serine residue associated to a histidine and a glutamate to compose the so-called catalytic triad. The structures of cholinesterases have been extensively studied in order to better understand their biochemical roles. The selective, reversible inhibition of AChE is important in the treatment of AD, allowing research on novel drugs. On other hand, their irreversible inhibition caused by nerve agents (Figure 1, Figure 2, Figure 3 and Figure 4), organophosphorus and carbamate pesticides, like chlorpyrifos **22** and aldicarb **23** (Figure 8) may be fatal, depending on the level of exposure and if not identified and immediately addressed [61,62,63,64,65,66,67,84].

**Scheme 2:**
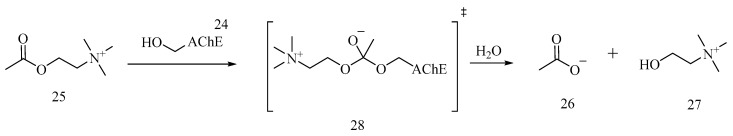
Hydrolysis of ACh through AChE.

AChE **24** catalyzes the breakdown of acetylcholine (ACh, **25**), into acetate **26** and choline **27** through a tetrahedral transition state **28** at the post-synaptic cleft, ending the action potential, in accordance with the proposal below (Scheme 2).

**Figure 9:**
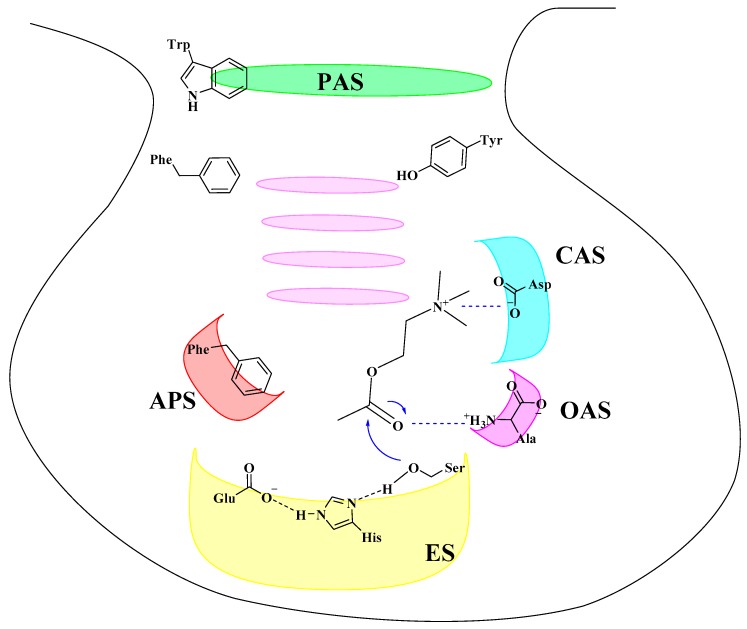
Active site of AChE [4] (ES: Estearic site, PAS: Peripheral Anionic Site, CAS: Catalytic Anionic Site, OAS: Oxianion Pocket, APS: Acyl Pocket Site, Ser: Serine, His: Histidine, Glu: Glutamate, Phe: Phenylalanine, Asp: Aspartate, Ala: Alanine, Trp: Tryptophan, Tyr: Tyrosine) Reproduced with permission.

A schematic representation of different sites of AChE is shown in Figure 9 [4,8,61,62,63,64,65,66,67]. The esteratic site (catalytic site, ES), is responsible for the hydrolysis reaction through the triad serine-histidine-glutamate (whose position in the enzyme primary structure may slightly vary across species, as cholinesterases are much conserved enzymes). Also relevant is the peripheral anionic site (PAS) which is formed by aromatic amino acid residues. There are studies correlating PAS and the occurrence of β-amyloid proteins (Aβ 1–42). These insoluble oligopeptides together with tau tangles are related to the onset of AD. PAS and its aromatic region assist cationic (or electrophilic) substrates to take the right direction towards the catalytic gorge. The catalytic anionic site (CAS) contains aromatic amino acid residues, like tryptophan and phenylalanine, which interacts with the cationic motif of the substrates, and helps to bring the ester motif of substrates close to the catalytic ES. The oxyanion pocket (OAS) helps to stabilize the tetrahedral transition state (**28**) during ACh hydrolysis. Finally, the acyl pocket site (APS) is the point where the difference between AChE and BChE may be assessed, with AChE having a smaller APS than BChE, determining the size of the substrate to be hydrolyzed [61,62,63,64,65,66,67,85,86,87,88,89,90,91,92,93].

## 4. The Inhibition of Acetylcholinesterase

As discussed, AChE is the enzyme involved in the breakdown of ACh **25**, the parasympathetic neurotransmitter, into choline **26** and acetate **27**, which are reuptaken for de novo synthesis of the neurotransmitter. Therefore, AChE is a pharmaceutical target, as its inhibition provokes an increase of neurotransmitter concentration at the post-synaptic cleft, leading to exacerbated cholinergic response over the nerve structures that require parasympathetic stimulation, such as neuromuscular junctions. This outcome may be positive, as in the case of treatment of diseases where the transitory, reversible inhibition of AChE located in the central nervous system (CNS) delivers valuable response, e.g., AD, or negative, when toxic chemicals, such as nerve agents (Figure 1, Figure 2, Figure 3 and Figure 4) or organophosphorus and carbamate pesticides (Figure 8), cause irreversible inhibition at neuromuscular junctions, which may be life-threatening [53,54,94,95,96,97,98,99,100,101,102,103].

**Scheme 3:**
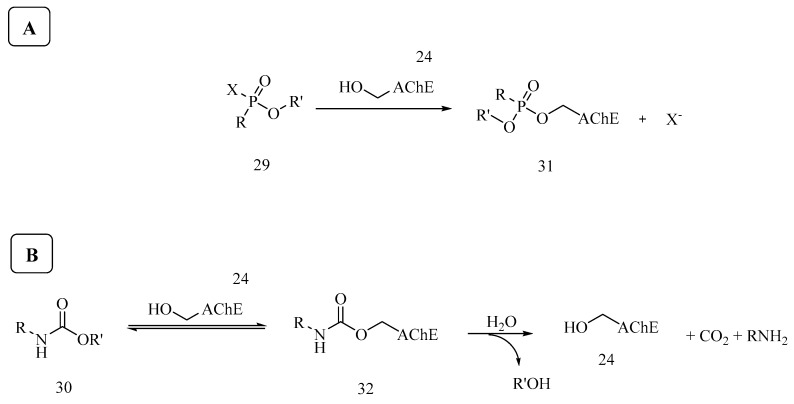
Reactions of AChE with different inhibitors.

Proposed reactions between AChE and inhibitors are represented in the Scheme 3. Inhibitors have an electrophilic site that reacts with the nucleophilic hydroxyl of the serine residue at the catalytic triad. The bond is then formed by displacement of a leaving group. In case of irreversible AChE inhibitors, like classic nerve agents (G- and V-Series), organophosphorus compounds **29**, the leaving group is either a halogen (fluoride in GB **2** and GD **3**) or other suitable entity (cyanide in GA **4**, Scheme 3A). In the case of carbamates of clinical use **30**, usually reversible AChE inhibitors (exemption given to the toxic pesticides and “Novichok” carbamates described previously in this text), an alcohol **31** is displaced (Scheme 3B).

## 5. Reversible Inhibition of AChE: A Tool for Treatment of AD

It is reckoned that ACh is involved in the cognition mechanism. Therefore, increased levels of this neurotransmitter may contribute to memory improvement. Based on this knowledge, the selective inhibition of AChE at the CNS has been used for treatment of AD, a multifactorial disease that causes affected people to suffer memory impairment and progressive neurodegeneration, which is not only fatal, but also a burden to the health system and people involved in patient care [100,101,102,103,104,105,106,107].

**Figure 10:**
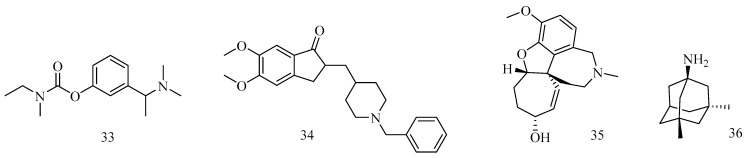
Currently approved drugs for treatment of Alzheimer’s disease (AD).

Currently, there are only four drugs available to ameliorate the memory loss and other symptoms related to AD. Nonetheless, they can only act in a certain level of impairment and are unable to halt the progression of the pathology. Consequently, knowledge on the different mechanisms that can contribute to the onset of AD and discover of the early neurological, biochemical, and behavioral changes, useful for the development of a more efficient treatments. Three of these compounds act as AChE inhibitors, rivastigmine (a carbamate, **33**), donepezil (a benzylpiperidine derivative, **34**), and galantamine (an alkaloid of natural occurrence, **35**). The fourth compound is memantine (an adamantane derivative, **36**), which affects other receptors (glutamatergic, serotoninergic, acetylcholine nicotinic receptors) [107,108,109,110,111,112,113,114,115,116,117,118,119,120,121,122,123,124,125,126,127]. These compounds are shown in Figure 10.

Rivastigmine is regarded to react with the serine residue of the ES present in AChE and BChE (as shown in Scheme 3), whereas donepezil can act in both CAS and PAS of AChE. These actions increase the synaptic levels of ACh, rendering positive cognitive response of the patient. In case of donepezil, action on PAS is further regarded to reduce the biosynthesis of insoluble β-amyloid oligopeptides that bring about inflammatory response that contribute to the neurodegeneration characteristic of AD. Galantamine acts simultaneously as an AChE inhibitor and allosteric modulator of nicotinic receptors, increasing the affinity for ACh [114,115,116,117,118,119,120,121,122,123,124,125,126,127,128]. Research in progress on novel AD drugs has covered different disciplines and been focused on further development of AChE inhibitors, beta-secretase 1 (BACE-1) inhibitors, serotonin 5-HT_4_ receptor inhibitors and PROTAC (PRO*teolysis* T*argeting* C*himeras*) compounds, among other approaches [129,130,131,132,133,134,135,136,137,138,139,140,141,142,143,144,145,146].

The use of carbamates as drugs is based on the comparison of hydrolysis rates of complexes between AChE and its natural substrate ACh and those yielded by reaction with carbamates (which reversibly carbamoylate the ES; Scheme 3B) which usually show lower rates [108,118,119]. Other important uses for AChE reversible inhibitors are the treatment of dementia related to Parkinson’s disease, and glaucoma and myasthenia gravis.

**Figure 11:**
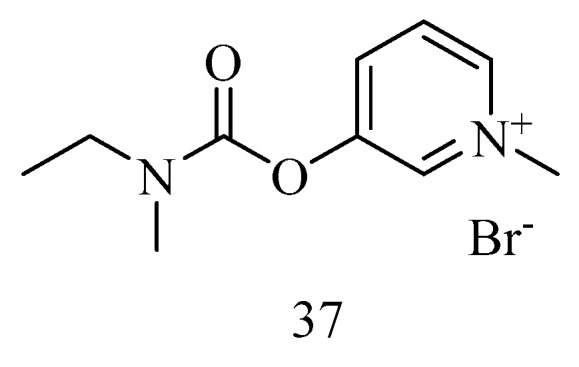
Pyridostigmine bromide.

Other carbamate that acts as AChE reversible inhibitor is pyridostigmine **37** (Figure 11), a drug used for treatment of myasthenia gravis, which has been used as protective measure for those working in places where nerve agents might have been deployed, as during Gulf War (1991). Overdose of pyridostigmine has been studied as a possible cause of the “Gulf War Syndrome” [109,110,147,148,149].

## 6. Irreversible Inhibition of AChE: The Chemistry of Nerve Agents

**Figure 12:**
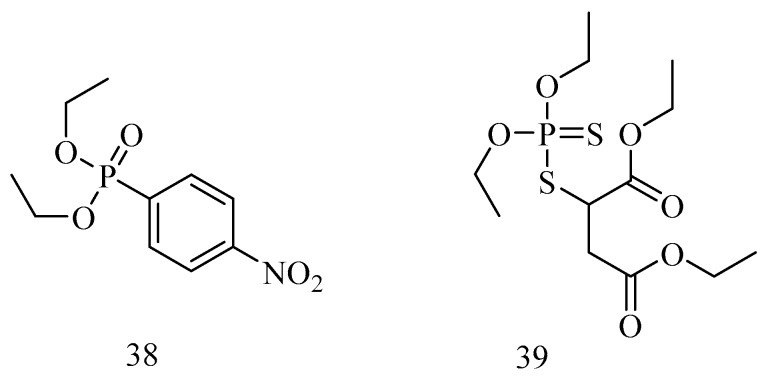
Organophosphorus pesticides paraoxon and malathion.

As expected, the irreversible AChE inhibition may result in life-threatening effects, according to the level of exposure and resources available to treat affected victims. The overstimulation may trigger a series of symptoms represented by the acronym *SLUDGEM* (**S**alivation, **L**achrimation, **U**rination, **D**efecation, **G**astrintestinal disturbs, **E**mesis, **M**yosis, and **M**uscle spasms) which illustrates the clinical effects. Acute exposure may be fatal due to the respiratory failure resulting from the continuous stimulation of neuromuscular junctions at diaphragm, as described earlier in this paper [6,14,19,20,53,54,150]. Classic (G- and V-Series) and “Novichok” nerve agents (Figure 1, Figure 2, Figure 3 and Figure 4) listed in the CWC in its Schedule 1, Part A, are example compounds. Structurally related to nerve agents, some pesticides, such as chlorpyrifos **22** and aldicarb **23** (Figure 6), paraoxon **38** (Figure 12) (used in many studies for development of antidotes towards cholinesterase inhibitors), and malathion **39** (Figure 12), a compound still used as aerosol to combat arboviruses vectors in developing countries (Figure 12) [3,4,5,6,8,19,45,46,50,53,54,151,152,153,154].

**Figure 13:**
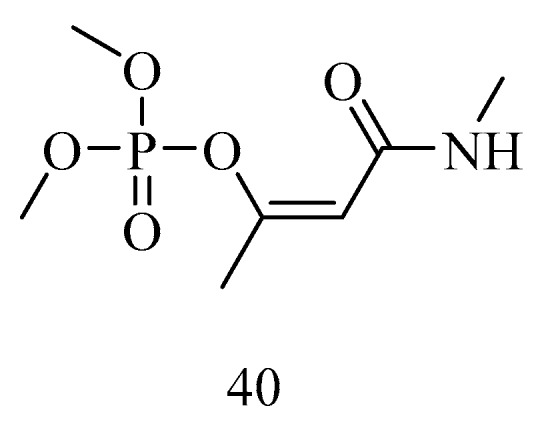
Monocrotophos.

The toxicity related to organophosphorus compounds may be explained through the high affinity of phosphorus and oxygen, with higher energy bonds, 335 and 544 kJ/mol, for P-O and P=O bonds, respectively [155]. Poisoning and environmental contamination by organophosphorus pesticides poses a serious public health challenge in countries with lack of control of these substances. Nonetheless, occupational poisoning is not the sole problem. Cases of pesticide use in suicides are also known. The World Health Organization (WHO) estimated more than 200,000 casualties per year by poisoning with organophosphorus pesticides in developing countries. Recently in India, more than 20 children died after eating a meal prepared with oil stored in monocrotophos-contaminated bottles (**40**, Figure 13) [156,157]. As an alternative, many countries have adopted neonicotinoids as insecticides, which are less toxic to mammalians and birds. However, they are under scrutiny due to toxicity for pollinator insects [158,159].

**Scheme 4:**
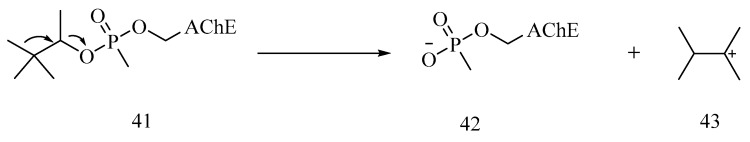
Aging of AChE-GD adduct.

Depending on the AChE adduct formed with organophosphorus compounds (**31**, Scheme 3A), the displacement of the O-alkyl moiety can lead to enzymatic aging. The generated phosphonate anion **41** is stabilized by the protonated histidine imidazole moiety located at the catalytic ES, affecting the antidote efficacy. Adduct of GD with AChE **42** quickly undergoes such process (Scheme 4) [160,161,162,163,164,165,166,167].

**Scheme 5:**
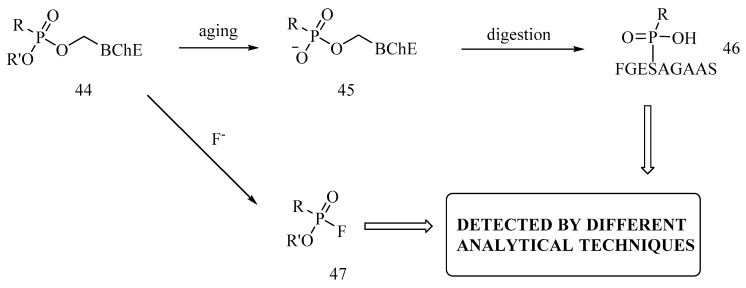
Digestion and fluoride reactivation products from biological matrices.

The knowledge on the reaction between nerve agents and cholinesterases may be useful to identify exposure to such toxic chemicals. In blood and plasma samples, nerve agents may be found in their hydrolyzed forms, mainly alkylphosphonic acids, but also as adducts with BChE **44**. If this adduct is aged **45**, its digestion yields a phosphylated nonapeptitde **46** (FGESAGAAS, A: Alanine, E: Glutamate, F: Phenylalanine, G: Glycine, S: Serine) that can be used as proof of exposure to nerve agents. Nevertheless, if the enzyme is not aged **44**, treatment with fluoride ions enables a de novo synthesis of the organophosphorus compound **47**. These compounds can be successfully detected by chromatographic and spectrometric techniques (Scheme 5). This reaction between BChE and neurotoxic CWC Schedule 1A not only highlights the importance of this enzyme as a biomarker, but also illustrates its potential use as a bioscavenger. Therefore, it can be a potential prophylactic measure by reacting in stoichiometric manner with nerve agents [168,169,170,171,172,173,174,175,176,177,178,179,180,181].

## 7. Rescuing Cholinesterases: Antidotes towards Nerve Agents

**Figure 14:**
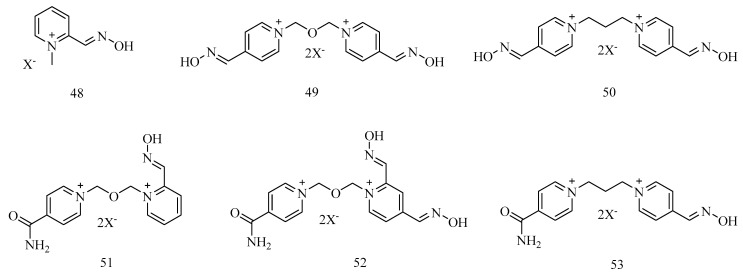
Oximes clinically used as antidotes for organophosphorus poisoning. X = chloride, mesylate or iodine for 48, chloride for 49, bromide for 50 and 53, chloride or mesylate for 51 and 52.

**Scheme 6:**
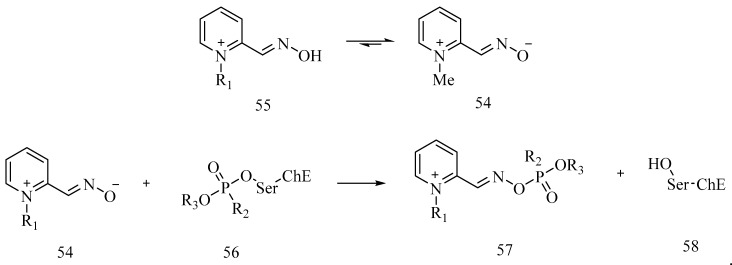
Reactivation of AChE through pyridinium oximes.

In order to rescue organophosphorus-inhibited AChE and BChE, appropriate antidote therapy must be promptly employed. Rapid response is required to lessen the risk of neurological damage and even death. Antidotes usually are a mixture of three different compounds, an enzyme reactivator (to remove the organophosphorus from catalytic ES), an anticholinergic agent (to counteract the effects of the higher concentration of the neurotransmitter), and an anticonvulsant (to control seizures). Up to date, pyridinium oximes have been clinically used as AChE reactivators, pralidoxime **48**, obidoxime **49**, trimedoxime **50**, HI-6 **51**, HLö-7 **52** and K027 **53** are representative compounds (Figure 14) [4,182,183,184,185,186]. At the physiological pH oximes afford oximates. These nucleophiles reactivate cholinesterases through a nucleophilic attack on the phosphorus atom, releasing the hydroxyl serine residue. Scheme 6 depicts the reaction of a pyridinium oximate **54** and an organophosphorus-inhibited cholinesterase **56** [4,99,160,161,185,186,187,188,189,190,191,192,193].

**Figure 15:**
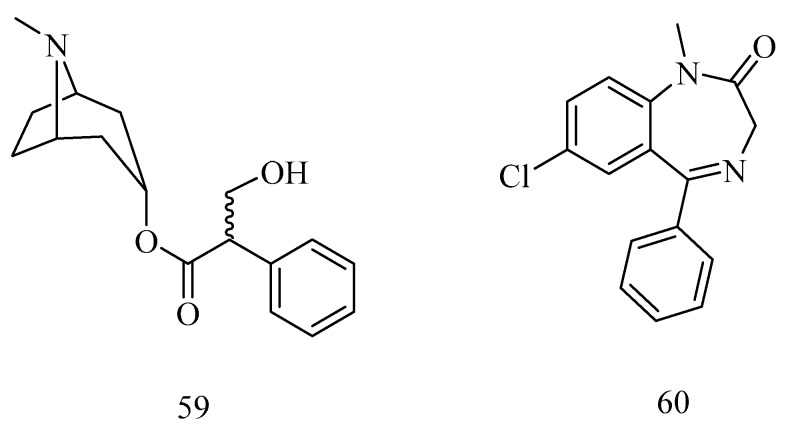
Atropine and diazepam.

Marketed kits also contain atropine **59** as anticholinergic agent, and an anticonvulsant, such as diazepam **60** (Figure 15). Seizures may be related to the excitotoxicity that takes place due to unbalanced levels of glutamate and γ-aminobutyric acid (GABA) in brain in the course of AChE inhibition. The use of glutamate antagonists and GABA agonists may be a valuable therapeutic approach [4,194,195,196,197].

Due to the cationic profile of all available antidotes, permeation into the blood-brain barrier (BBB) hinders effective treatment. Additionally, an efficient “universal antidote’ towards all cholinesterase inhibitors is not yet available [198]. To tackle these limitations, development of more lipophilic compounds and novel routes of administration of antidotes have been extensively studied [4,199,200,201,202,203,204,205].

## 8. Final Remarks

Cholinesterases are constitutive enzymes, essential to the correct performance of the parasympathetic nervous system (PNS). The chemistry of AChE inhibition and aging in the context of the contemporary issue of terrorism by chemical warfare is an attractive conduit for chemical education and for dual use, military and civilian research work. AChE has a pivotal role in memory and learning, therefore increased knowledge on AChE function may facilitate the development of further, more effective therapies towards neurodegenerative diseases, like AD. Besides the varied conceptual background in Chemistry that it demands, the subject is also multidisciplinary, since it is related to pharmacology, toxicology, medicinal chemistry, international politics, health, environmental and forensics sciences, as well ethical issues.
